# Chemiresistive Gas Sensors for the Detection of *Listeria monocytogenes* Metabolite: Recent Progress and Challenges

**DOI:** 10.3390/bios16080438

**Published:** 2026-08-13

**Authors:** Bingxi Feng, Jing Wei

**Affiliations:** 1 College of Environment and Life Sciences, Weinan Normal University, Weinan 714099, China; 2 Institute of Analytical Chemistry and Instrument for Life Science, The Key Laboratory of Biomedical Information Engineering of Ministry of Education, School of Life Science and Technology, Xi’an Jiaotong University, Xi’an 710049, China; 3 Key Laboratory for Ecology and Environment of River Wetlands in Shaanxi Province, Weinan 714099, China

**Keywords:** *Listeria monocytogenes*, chemiresistive gas sensor, volatile organic compounds, 3-hydroxy-2-butanone, food safety monitoring

## Abstract

*Listeria monocytogenes* (LM), one of the most virulent foodborne pathogens, poses a serious threat to public health due to its strong environmental adaptability and high pathogenicity. Rapid, sensitive, and real-time detection of LM is of great importance. Chemiresistive gas sensors have attracted enormous attention in LM detection owing to their advantages of low cost, simple structure, fast response, and easy miniaturization, which can achieve indirect detection of LM by recognizing its specific metabolic volatile organic compounds. This review summarizes the recent progress in chemiresistive gas sensors for the detection of LM metabolites. First, the metabolic characteristics of LM and the typical volatile organic compound (3-hydroxy-2-butanone) as its characteristic biomarker are introduced. Then, the performance and sensing mechanisms of different types of chemiresistive gas sensors for LM metabolite detection are summarized and elaborated systematically. The application of chemiresistive gas sensors for the detection of actual samples and the progress in the design of related detection devices are introduced. Finally, the current challenges faced by chemiresistive gas sensors in LM metabolite detection and their future development prospects are discussed. This review provides a comprehensive reference for the research and practical application of chemiresistive gas sensors in *Listeria monocytogenes* detection.

## 1. Introduction

*Listeria monocytogenes* (LM) is one of the most prevalent foodborne pathogens. It has a high mortality rate and great environmental adaptability, and poses a great threat to public health [1,2,3]. LM can survive and grow in extreme environments, such as refrigeration temperatures, acidic conditions, and high-salt concentrations. Thus, it occurs during the whole food processing, storage, and distribution process [4]. The intake of contaminated food, such as milk products, meats, seafood, and fresh produce, can cause severe listeriosis. In pregnant women, newborns, the elderly, and immunocompromised individuals, the mortality rate of LM infection can reach up to 20–30% [5]. The detection methods of LM include polymerase chain reaction, enzyme-linked immunosorbent assay, and loop-mediated isothermal amplification [6,7,8,9,10]. In addition, emerging platforms such as microfluidic-based technologies have attracted increasing attention in biological analysis owing to their advantages in miniaturization, rapid response, and integration capability [11]. However, many existing methods still require complex sample preparation, sophisticated instruments, or specialized personnel, limiting their application for rapid and real-time monitoring of LM. Therefore, there is an urgent need for establishing a rapid, real-time detection technology for LM.

Recent research shows that certain volatile organic compounds (VOCs) are released during the metabolism of microorganisms. Thus, bacteria can be indirectly detected by detecting these VOCs [12,13,14]. Compared with direct identification of bacteria, the analysis of VOCs has a number of advantages, such as non-contact sampling, minimal sample treatment, and adaptability to continuous detection. Gas chromatography-mass spectrometry (GC-MS)-based metabolomic studies have revealed that LM produces diverse volatile metabolites, and 3-hydroxy-2-butanone (acetoin, 3H-2B) has been repeatedly found to be the most representative biomarker due to its relatively high abundance, favorable volatility, and reproducible synthesis in bacterial growth [15,16]. Thus, developing high-performance 3H-2B gas sensors for the detection of LM in food is critical.

Chemiresistive gas sensors have received attention due to their low manufacturing cost, fast response, high sensitivity, and compatibility with the miniaturization of electronic devices [17,18,19]. More importantly, with the recent advances in material engineering, such as morphology regulation, heteroatom doping, noble metal functionalization, and heterojunction construction, the performance of chemiresistive sensors toward 3H-2B has been significantly enhanced with high detection speed of trace LM biomarkers in complex environments [12,13,20,21]. The incorporation of the gas sensors with micro-electromechanical systems (MEMS) technology, wireless communication, and intelligent sensing systems has further facilitated their application in portable food safety monitoring [22,23].

Although notable advancements have been achieved in the development of chemiresistive gas sensors for the detection of LM metabolites, there is a lack of a comprehensive review on this emerging research field. In this work, we first review the metabolic properties of LM and its volatile biomarkers and then introduce recent advances in the development of chemiresistive gas sensors from the perspectives of sensing materials, sensing mechanisms, and performance improvements. Finally, the actual applications, current challenges, and prospects of chemiresistive gas sensors for the detection of LM metabolites are discussed to provide insights for the development of next-generation intelligent sensing platforms.

## 2. Metabolic Volatile Biomarkers of *Listeria monocytogenes*

*Listeria monocytogenes* is a facultative intracellular Gram-positive foodborne pathogen capable of surviving under a wide range of environmental conditions, including refrigeration temperatures, acidic environments, and high-salt foods. The significant environmental adaptability is mainly due to its flexible metabolic network, which allows it to use diverse carbon sources for maintaining growth [2,6]. During carbohydrate metabolism, glucose is processed into pyruvate through glycolysis, where the pyruvate serves as a key metabolic intermediate and is further processed into different fermentation products, e.g., lactate, 2,3-butanediol, acetate, ethanol, and 3H-2B, depending on environmental conditions and metabolic requirements [24]. Apart from providing energy production and ensuring the intracellular redox balance, these metabolic reactions play indispensable roles in bacterial stress adaptation. Specifically, recent studies have demonstrated that LM has the capability of reprogramming its core carbon metabolism for the production of neutral metabolites, e.g., 3H-2B, under acidic environmental conditions, thus mitigating the intracellular acidification and promoting environmental tolerance. Therefore, the metabolic activities of LM are accompanied by the continuous generation of VOCs, which can serve as volatile biomarkers for LM detection.

The VOC profile of LM is composed of various alcohols, ketones, aldehydes, esters, organic acids, and sulfur compounds [15,16]. With the development of headspace solid-phase microextraction coupled with GC-MS, more and more studies have been undertaken to characterize these volatile metabolites, and have shown that the composition is dependent on bacterial strains, growth phase, culture medium, and environmental conditions. However, ketones and alcohols have consistently been found to be the predominant VOCs produced during LM growth [24]. Metabolic comparisons have shown that the volatile profile of LM is different from that of common foodborne pathogens such as *Escherichia coli*. These observations suggest that VOCs can be characteristic metabolic signatures for bacterial discrimination and serve as indirect detection strategies targeting bacterial metabolites.

**Figure 1:**
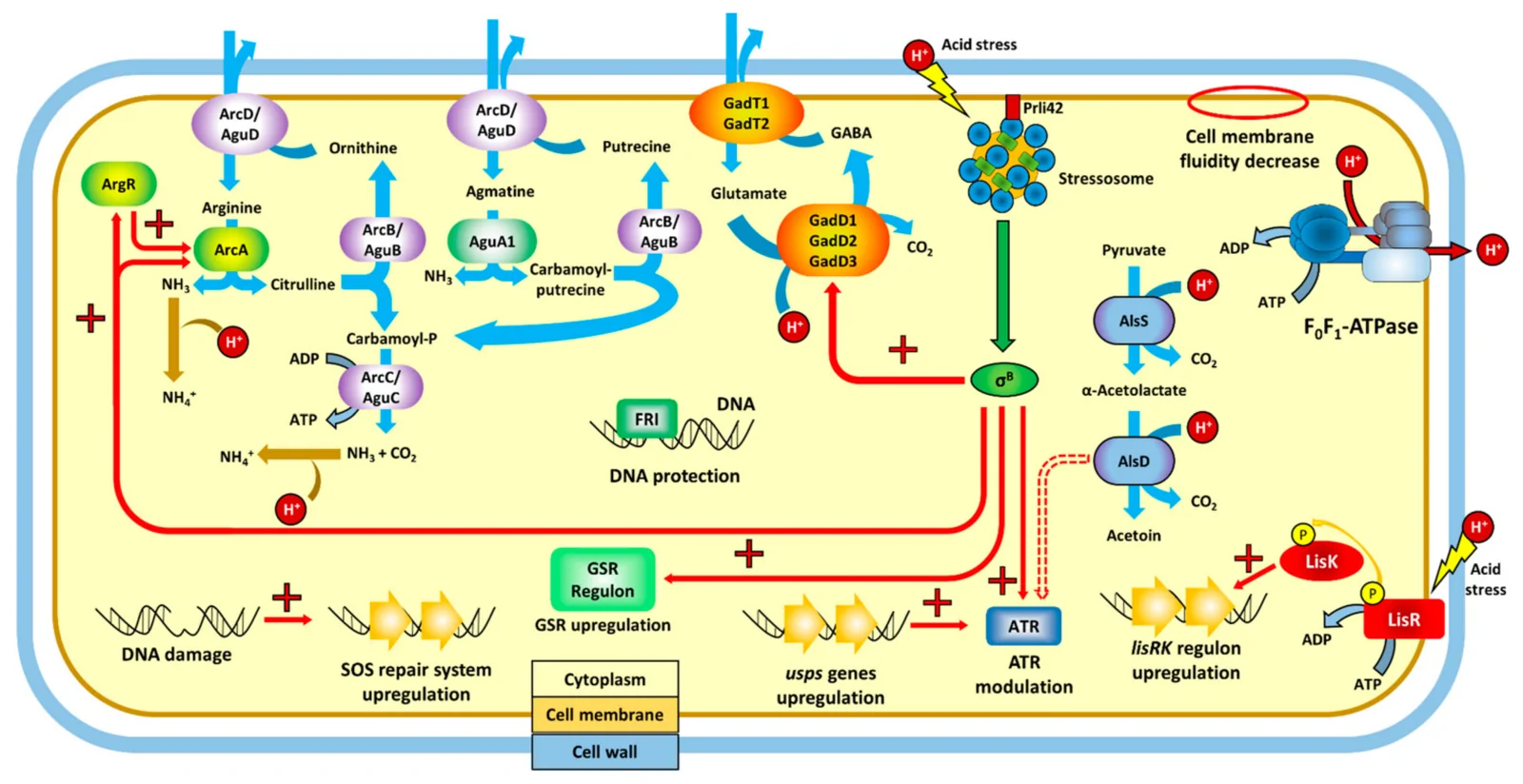
Schematic biosynthetic pathway of 3H-2B (acetoin) in *Listeria monocytogenes* [24]. Reprinted from Ref. [24] under the terms of the Creative Commons Attribution (CC BY 4.0) License.

Among the reported VOCs, 3-hydroxy-2-butanone has drawn the most attention as a typical biomarker for the detection of LM due to its relatively high abundance, good volatility, and consistent production during bacterial growth (Figure 1). 3H-2B is synthesized through the butanediol fermentation pathway, where pyruvate is first transformed into *α*-acetolactate by α-acetolactate synthase (AlsS) and then decarboxylated into 3H-2B by *α*-acetolactate decarboxylase (AlsD) [24]. Under appropriate redox conditions, 3H-2B can be further reduced to 2,3-butanediol by butanediol dehydrogenase. As a neutral metabolite, 3H-2B contributes to intracellular pH homeostasis and redox balance, allowing LM to better withstand acid stress while maintaining metabolic activity. More importantly, multiple GC-MS-based metabolomic studies and subsequent gas-sensing investigations have consistently identified 3H-2B as the most representative volatile biomarker of LM [15,16]. This discovery has shifted the focus of LM detection from direct bacterial identification to the selective monitoring of a characteristic volatile metabolite. Consequently, chemiresistive gas sensors capable of selectively detecting trace levels of 3H-2B have emerged as a promising strategy for rapid, non-destructive, and on-site monitoring of LM contamination in food systems [12,13,14,23,25].

## 3. Chemiresistive Gas Sensors for Detecting *Listeria monocytogenes*

3H-2B was identified as one of the most representative biomarkers released during the metabolism of LM. As such, LM can be indirectly detected by monitoring 3H-2B. The sensing response of chemiresistive gas sensors is mainly derived from the interaction between target gas molecules and sensing materials. In MOS-based sensors, oxygen molecules are first adsorbed on the surface and capture electrons, forming reactive oxygen species. The reaction between VOC molecules and adsorbed oxygen species leads to charge transfer and changes in sensor resistance [26]. For n-type MOS sensors, the resistance usually decreases after exposure to reducing gases, while p-type sensors show an opposite response due to different charge carriers [27,28]. Chemiresistive gas sensors have drawn particular attention because of their simple device architecture, fast response, high sensitivity, inexpensive fabrication, and compatibility with miniaturized and portable sensing systems. More importantly, their sensing performance can be tailored effectively via rational material engineering, allowing for high-performance detection of trace volatile biomarkers. Considerable efforts have been given to developing high-performance chemiresistive sensing materials for 3H-2B detection in recent years. Some representative strategies include the use of single-component metal oxide semiconductors (MOS), heteroatom doping, noble metal modification, and heterojunction construction. They can enhance gas adsorption, catalytic activity, charge transport, and interfacial reaction kinetics through different mechanisms. This section systematically summarizes the material engineering strategies and discusses their sensing performance and sensing mechanism.

### 3.1. Single-Component Metal Oxide Semiconductors

MOS constitutes a representative class of chemiresistive gas sensing materials. They possess a series of unique characteristics such as inexpensive fabrication, an easy synthesis process, excellent chemical stability, and high microelectronics manufacturing compatibility [29,30,31,32]. The sensing mechanism is based on interfacial redox reactions. The contact between the adsorbed oxygen groups and volatile compounds upon sensing of the target gas leads to a significant variation in the electrical resistance of the material [33,34]. This variation makes it possible to quantitatively translate chemical information into a measurable electrical response [35,36,37]. The MOS materials provide highly tunable structure, controllable micro-morphology, and rich surface reactive sites. Such properties allow optimized gas sensing performance, making the MOS-based sensors highly competitive for monitoring VOCs.

**Figure 2:**
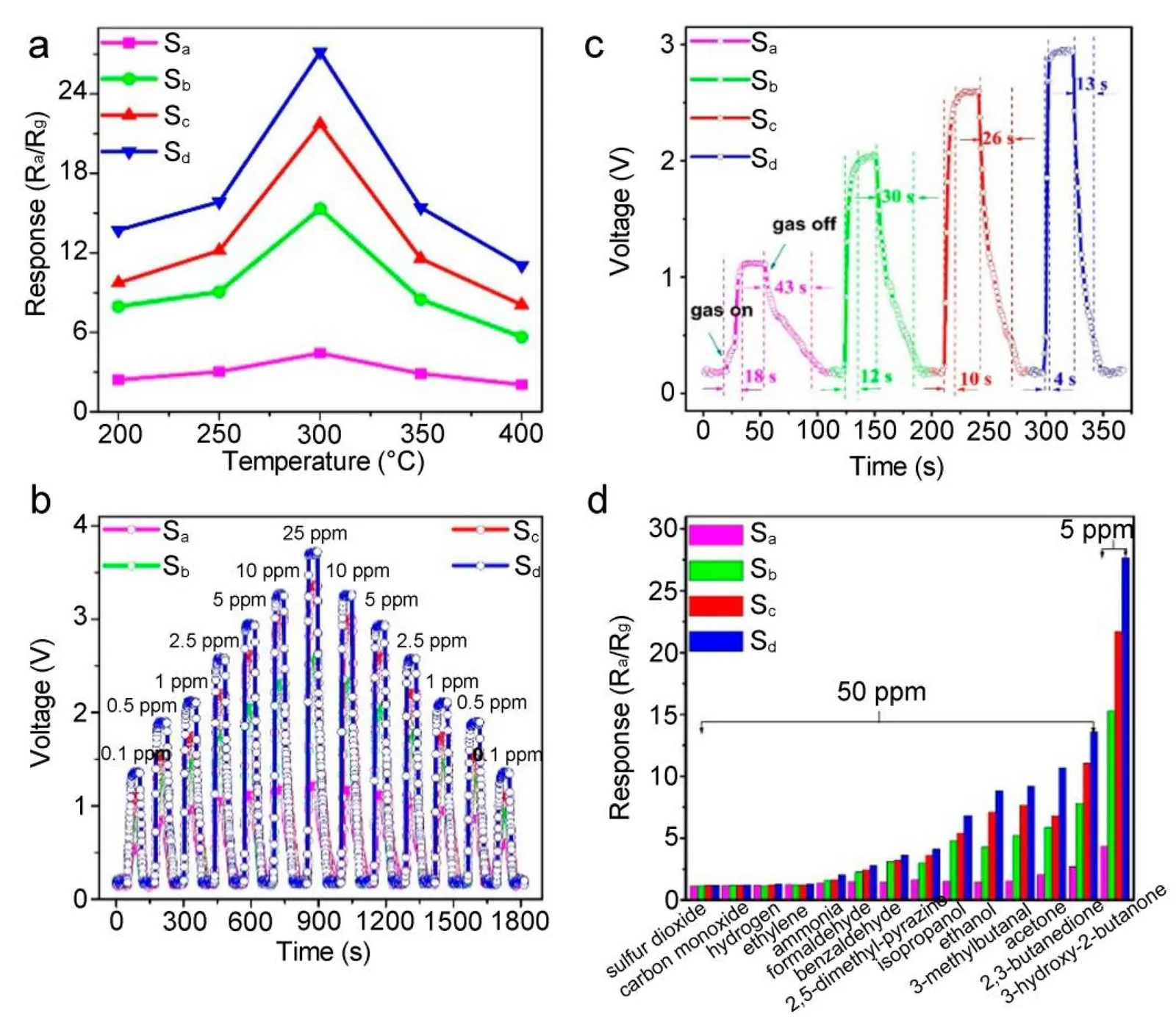
(**a**) Responses toward 5 ppm of 3H-2B across various operating temperatures. (**b**) Responses toward different concentrations (0.1–25 ppm) of 3H-2B at 290 °C. (**c**) Response and recovery curve toward 5 ppm 3H-2B. (**d**) Selectivity of the sensor toward 5 ppm 3H-2B compared with 50 ppm interfering gases. (Sa, Sb, Sc, and Sd: WO_3_ particles, WO_3_-PEO_117_-b-PS_297_, WO_3_-PEO_117_-b-PS_232_, and WO_3_-PEO_117_-b-PS_186_) [12]. Adapted with permission from Ref. [12]. Copyright 2017 American Chemical Society.

Zhu et al. reported the first indirect detection of LM based on its unique volatile biomarker using a chemiresistive metal oxide semiconductor [12]. They synthesized a highly organized and mesoporous crystalline WO_3_ sensing material for the selective detection of 3H-2B. The interconnected pore mesoporous structure increased the specific surface area and supplied rich accessible active sites, while the continuous crystalline skeleton guaranteed effective electron transmission across the entire sensing layer. The WO_3_-based sensor demonstrated high response (56.1@25 ppm) to trace 3H-2B, short response and recovery time (4 s and 13 s), and great selectivity against other typical microbial VOCs (Figure 2). The sensor successfully identified LM by measuring the liberated volatile biomarker. The excellent sensing performance is due to the synergistic effect of the ordered mesoporous structure and the crystalline architecture. The rich pore network resulted in the fast diffusion of 3H-2B molecules and rich adsorption sites for the surface reaction. Meanwhile, the crystalline framework suppressed carrier scattering and facilitated carrier migration in the adsorption–desorption process. The oxidation of adsorbed 3H-2B on the sensing interface of metal oxide caused a significant modulation of the electron-depleted layer, leading to a significant resistance change. This study successfully demonstrates that single-component metal oxide semiconductors can detect LM via 3H-2B.

Besides n-type semiconductor metal oxides such as WO_3_, p-type semiconductor metal oxides are also utilized to detect 3H-2B. Traditional NiO usually has low gas diffusion efficiency and slow kinetic surface reactions. To overcome these drawbacks, Zhu et al. adopted a morphology engineering approach for the synthesis of multichannel pathway-enriched mesoporous NiO nanocuboids (M-NiO) [38]. The unique nanocuboid architecture possesses interconnecting mesoporous channels with a highly crystalline structure. The structure offers an abundance of active sites and promotes faster transport of analyte molecules across the sensing layer. Thus, excellent sensing performance was exhibited by this M-NiO-optimized sensor. When operating at an optimal temperature of 120 °C, it achieved a high response value of 302 towards 50 ppm of 3H-2B. In addition, fast response and recovery time of 49 s and 52 s were shown by this sensor. It has high selectivity against typical interfering VOCs. The long testing period has proved the stable performance of this sensor. Such excellent performance is attributed to the designed multichannel mesoporous design. With the interconnecting pore network, the diffusion pathway of gas is shortened to enable 3H-2B to quickly penetrate the sensing layer completely and boost the consumption of the active site on the surface. On the other hand, the high crystallinity of the NiO framework contributes to the boosted transport performance. The mesoporous structure also boosts the adsorption of oxygen, promoting more amounts of surface reactive oxygen species on the surface.

Several single-component metal oxide semiconductors have been used to detect 3H-2B. The sensing performances are listed in Table 1. For chemiresistive gas sensors, the response is generally determined based on the change in sensor resistance before and after exposure to target gases. Depending on the semiconductor type, the response is commonly defined as R_a_/R_g_ or R_g_/R_a_, where R_a_ represents the sensor resistance in air and R_g_ represents the resistance under target gas exposure. WO_3_ is the most extensively studied single-component metal oxide semiconductor owing to its excellent electronic characteristics, high catalytic activity for oxygen adsorption, and considerable affinity for oxygen-containing volatile compounds. However, the sensing performance of single-component metal oxide semiconductors is often limited by their intrinsic physicochemical properties, including the limited number of active sites, insufficient surface catalytic activity, and sluggish charge transport. To overcome these limitations, researchers have developed various material engineering strategies to improve the sensing performance.

### 3.2. Heteroatom Doping

Single-component metal oxide semiconductors display promising potential for sensing 3H-2B, but their performance is limited as described in the previous section. To overcome these shortcomings, ion doping has emerged as a promising strategy. The introduction of foreign metal ions into the crystal lattice modulates the local electronic configuration, the defect chemistry, and the surface adsorption properties of the metal oxides without disrupting the fundamental host structure [46,47,48,49]. In particular, appropriate dopants create a lattice distortion, optimize the formation of oxygen vacancies, enhance the chemisorbed oxygen species, and optimize the carrier transport [50,51,52,53]. These modifications enable the adsorption and subsequent oxidation of the target gas. These synergies can enhance sensitivity, improve selectivity, reduce the operating temperature, and speed up the response and recovery time. Accordingly, several doped metal oxide semiconductors have been developed for sensing 3H-2B, which presents an efficient strategy to enhance the performance of the chemiresistive gas sensors for LM.

**Figure 3:**
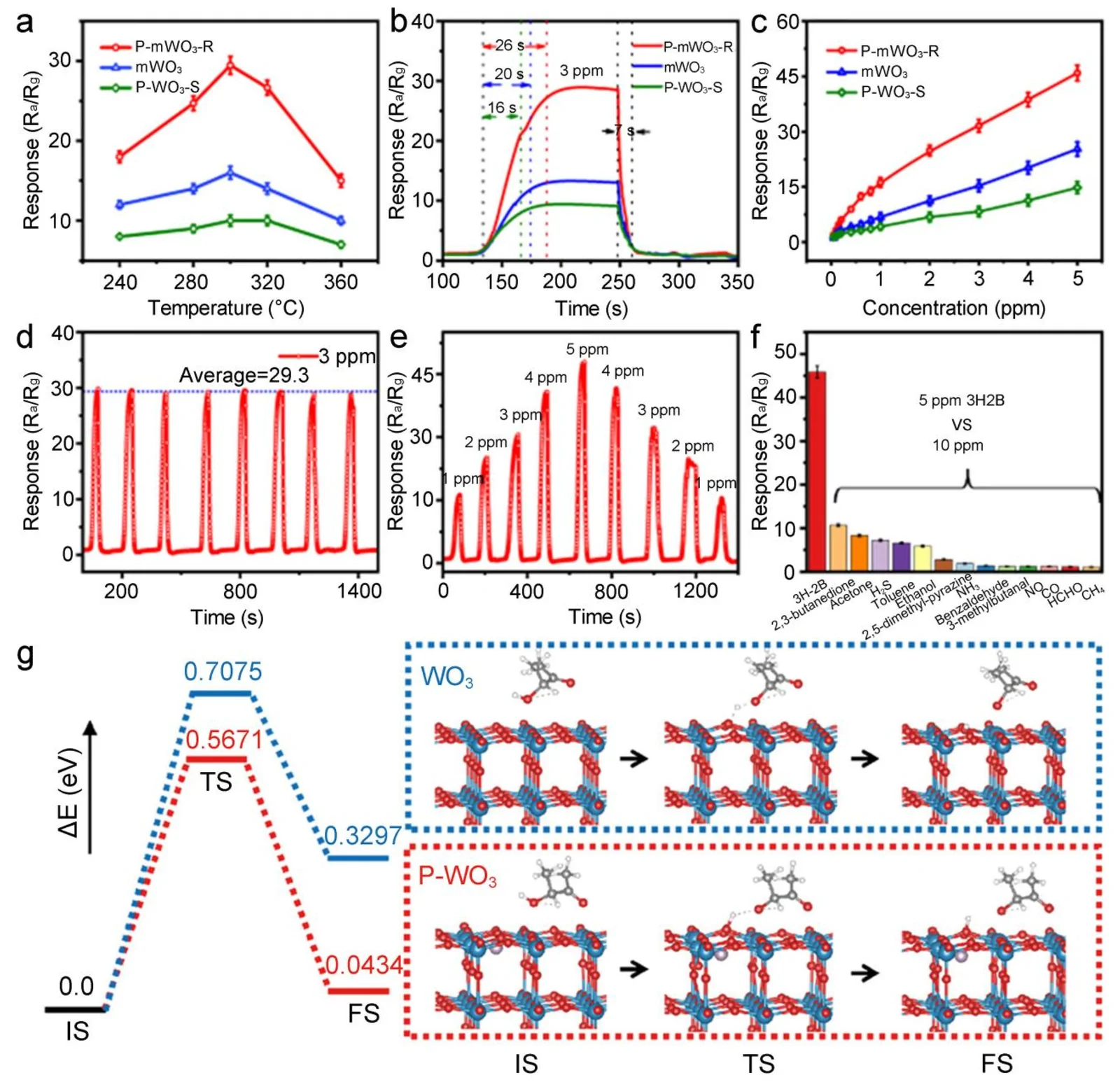
(**a**) Response–temperature curves of P-WO_3_-R spheres, mWO_3_, and P-WO_3_-S spheres sensors exposed to 3 ppm 3H-2B. (**b**) Response–recovery curve toward 3 ppm 3H-2B at 300 °C. (**c**) Response–concentration curves toward 3H-2B at 300 °C. (**d**) Response–recovery repeatability curves toward 3 ppm 3H-2B at 300 °C. (**e**) Cyclic response–recovery characteristics at 300 °C. (**f**) Selectivity performance of the P-mWO_3_-R sphere sensor at 300 °C. (**g**) Reaction energy profile for the surface dehydrogenation of 3H-2B on P-WO_3_ (FS: final state; TS: transformation state; IS: initial state) [13]. Adapted with permission from Ref. [13]. Copyright 2023 American Chemical Society.

Chen et al. prepared phosphorus-doped mesoporous WO_3_ microspheres (P-mWO_3_-R) with radially oriented channels using an alkaloid precipitation reaction method [13]. The prepared particles have a uniform particle size of about 180 nm. Taking advantage of these structural characteristics, the optimized sensor achieved a high response (29@3 ppm) with fast response and recovery time of 26 s and 7 s, respectively (Figure 3a–c). Also, it exhibited good long-term stability and excellent selectivity against the interfering VOCs (Figure 3d–f). This device was incorporated into a wireless monitoring module for real-time, remote detection of the LM in food samples, demonstrating good intelligent food safety surveillance potential. The superior device performance was explained by the synergistic effects of the defect engineering coupled with the structural optimization. Incorporating P changes the local coordination of the tungsten atoms, creating plenty of W^δ+^-O_v_ active sites as well as stimulating the formation of chemisorbed oxygen species, which speed up the electron transfer as well as the surface oxidation of 3H-2B. In addition, the radially aligned mesoporous channels decreased the gaseous diffusion path and optimized the utilization of active sites, which resulted in fast reaction kinetics. The electron paramagnetic resonance spectra and free energy profile proved the electronic regulation due to the phosphorus incorporation that reinforced the adsorption and activation of the 3H-2B molecules, explaining the remarkable enhancement in sensitivity and selectivity compared with the undoped mesoporous WO_3_ (Figure 3g).

Apart from single-element doping, co-doping with multiple heteroatoms has emerged as an effective strategy to enhance the performance of metal oxide semiconductors. This approach enables the synergistic regulation of both the crystal structure and surface electronic states. Hu et al. synthesized hierarchical Ce/P co-doped WO_3_ microstructures composed of nanoneedles (Ce/P-WO_3_) via a cluster-nuclei assembly strategy for the efficient detection of 3H-2B [54]. Compared with pristine WO_3_ and single P-doped WO_3_, the optimized Ce/P-WO_3_ sensor exhibited the highest performance. At an optimum operating temperature of 210 °C, it delivered a response of 7.2 toward 10 ppm of 3H-2B. The sensor also demonstrated high selectivity, with responses to 3H-2B approximately 3 to 5 times higher than those toward common interfering gases, such as ethanol, ammonia, and triethylamine. Its performance remained stable after 50 consecutive cycles and 32 weeks of storage, confirming excellent repeatability and long-term stability. This enhanced sensing performance is attributed to the synergistic effects of Ce/P doping and the hierarchical micro/nanostructure. Phosphorus incorporation generates abundant Lewis acid sites and oxygen vacancies. Simultaneously, Ce^4+^ integration into the WO_3_ lattice redistributes the local electronic structure and increases the adsorption strength for 3H-2B. The hierarchical architecture provides numerous exposed active sites and shortens the gas diffusion pathway, accelerating transport and surface reaction kinetics. MS analysis and density functional theory calculations revealed that Ce/P co-doping promotes the activation and oxidative decomposition of the analyte. It achieves this by increasing the concentration of reactive oxygen species and lowering the energy barrier for surface reactions, which leads to faster charge transfer and larger resistance modulation. These results demonstrate that synergistic multi-heteroatom doping is a promising strategy for improving the performance of chemiresistive gas sensors.

Heteroatom-doped metal oxide semiconductors have been reported to sense 3H-2B. Their sensing performances are summarized in Table 2. The electronic structure is regulated, the concentration of oxygen vacancies and chemisorbed oxygen species is boosted, and the adsorption and activation of target gas molecules are promoted, which result in improved sensitivity and selectivity of the sensing materials, with lower operating temperatures and faster response for most doped sensing materials than their non-doped counterparts.

### 3.3. Noble Metal Functionalization

Noble metal modification is widely employed to improve the sensing performance of chemiresistive gas sensors. The surface of metal oxides is decorated with catalytically active noble metals (such as Au, Pt, Pd, and Ag) to greatly increase surface reaction kinetics and charge transfer without significant modification to the host crystal structure. Such improvement is generally achieved by two main mechanisms: chemical sensitization and electronic sensitization. During chemical sensitization, the introduction of noble metal nanoparticles accelerates the adhesion/activation/catalytic oxidation of the target gas molecule [61,62,63]. During electronic sensitization, the noble metal oxide work function difference leads to interfacial electron redistribution [64,65,66], resulting in the formation of Schottky barriers and local depletion areas to boost the resistance modulation. Altogether, these synergistic mechanisms lead to reduced operating temperatures, short response/recovery time, and enhanced sensitivity and selectivity. Therefore, noble metal-modified metal oxide semiconductors constitute a promising material design option for the high-efficiency detection of 3H-2B.

**Figure 4:**
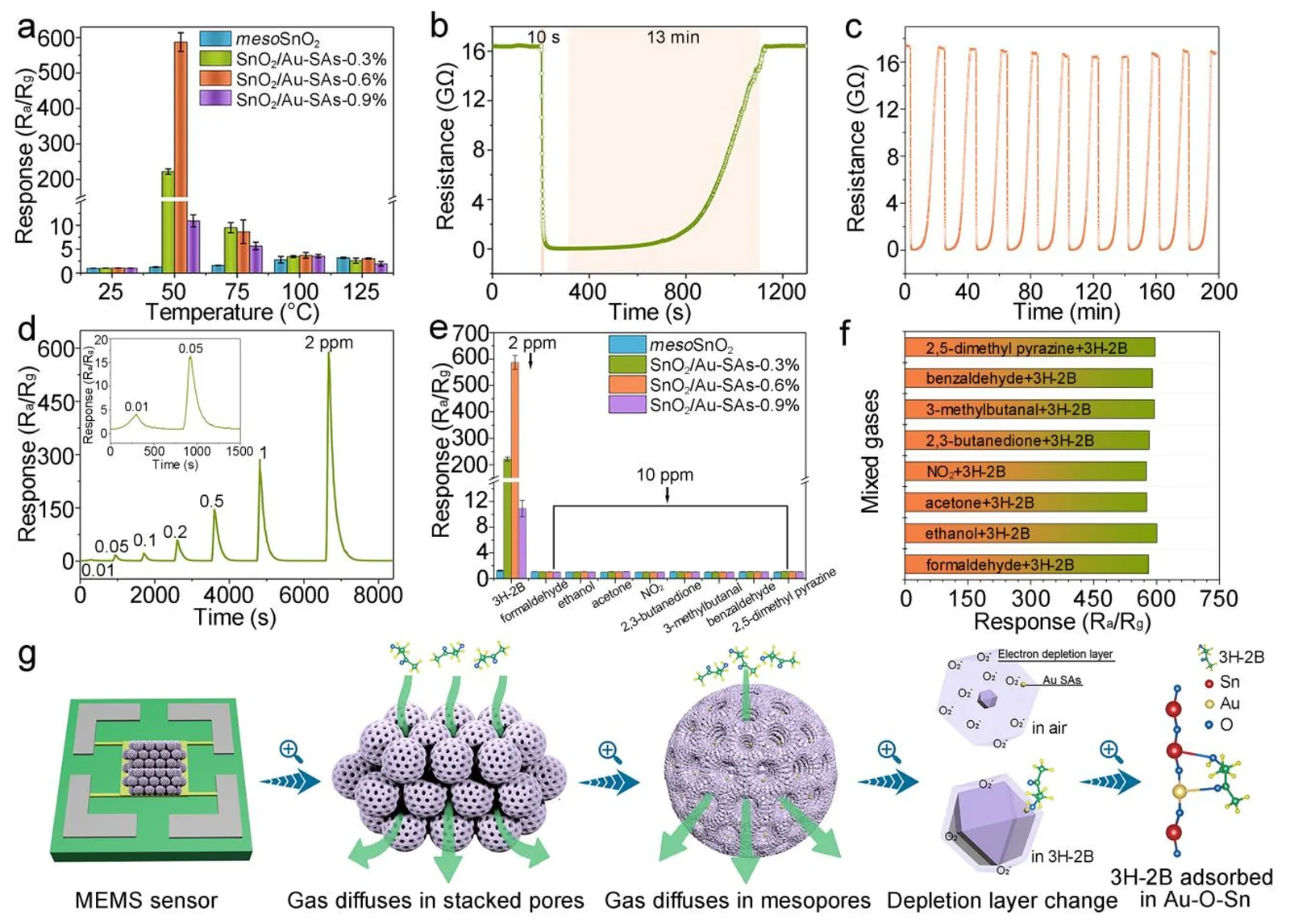
(**a**) Response–temperature curve toward 2 ppm 3H-2B. (**b**) Sensing kinetics, and (**c**) Operational stability toward 2 ppm 3H-2B at 50 °C. (**d**) Response–concentration characteristics of 3H-2B at 50 °C. (**e**) Gas-sensing selectivity at 50 °C. (**f**) Responses toward mixed gases of 3H-2B and other gases at 50 °C. (**g**) Schematic of the enhanced 3H-2B sensing mechanism on SnO_2_/Au-SAs [14]. Adapted with permission from Ref. [14]. Copyright 2024 American Chemical Society.

Among noble metals, Au has received much attention owing to its excellent catalytic performance and pronounced electronic interaction with semiconductor metal oxides. Feng et al. presented single-atom Au-functionalized mesoporous SnO_2_ nanospheres for detecting 3H-2B at low temperature [14]. Using Au as a single-atom catalyst rather than normal nanoparticles maximizes the utilization efficiency of the catalyst while maintaining the mesoporous framework of the SnO_2_ structure. Forming uniform nanospheres (~85 nm) with a large specific surface area offers plenty of active sites as well as gas diffusion channels. The optimized sensor showed an ultra-high response value (587.3) at 2 ppm 3H-2B at 50 °C, which is 183-fold higher than pristine mesoporous SnO_2_ (Figure 4a). The sensor obtained a sensitivity of 291.5 ppm^−1^, a low limit of detection (10 ppb), and a short response time (10 s) with high selectivity to typical interferant gases (Figure 4b–f). The sensor distinguished LM from *Escherichia coli*, *Salmonella*, *Staphylococcus aureus*, and *Streptococcus thermophilus* by tracing the volatile metabolites. This sensing material was fabricated into a MEMS-based wireless platform to realize real-time remote monitoring for intelligent food safety. The excellent capability is due to the synergistic effects of both single-atom Au and the mesoporous SnO_2_ structure. The uniformly distributed Au atoms generate abundant Au-O-Sn interfacial active sites, which improve the interaction with 3H-2B molecules, as well as accelerate the oxygen activation and catalytic oxidation process (Figure 4g). Meanwhile, the work function difference between Au and SnO_2_ induces interfacial electron redistribution that enhances the resistance modulation effect via electronic sensitization. The mesoporous framework reduces gas diffusion resistance while providing abundant active sites, thereby facilitating rapid surface reactions. Thus, the collaborative effects of chemical sensitization, electronic sensitization, and efficient gas diffusion help realize highly sensitive detection at significantly lower operating temperatures. This study manifests the merit of single-atom noble-metal engineering for low-power-consumption chemiresistive sensors.

In addition to the Au-based sensitization, Ag modification greatly boosted the performance of metal oxide semiconductors for 3H-2B detection. Wang et al. created an Ag-functionalized urchin-like WO_3_ MEMS sensor to detect LM contamination [67]. The surface of three-dimensional urchin-like WO_3_ microspheres was decorated with Ag nanoparticles and combined with MEMS sensors to obtain highly sensitive, low-power consumption. The optimized Ag/WO_3_ sensor showed the best performance with a response of 92.7 to 30 ppm 3H-2B at an operating temperature of 240 °C. In addition, the short response and recovery time was obtained as 14 s and 18 s; the sensor showed high selectivity against the main interfering gases and stable performance in multiple measurements. The excellent performance is owed to the effects of the Ag sensitization and the hierarchical urchin-like WO_3_ microstructure. The radially arrayed nanorods are formed into a porous three-dimensional framework with abundant exposed reaction sites and open diffusion channels, which provides for the fast transport of target molecules in the sensing layer. Meanwhile, Ag nanoparticles provide highly active catalytic sites, accelerating the adsorption and oxidation of 3H‑2B via chemical reactions. Since Ag has a higher work function than WO_3_, electrons are transported from the semiconductor to the metal, forming Schottky barriers at the metal-semiconductor interface. The electronic sensitization effect enlarges the thickness of electron depletion layer, thereby amplifying the resistance variation during exposure to 3H-2B. Therefore, the synergistic effects of the hierarchical microstructure, Ag-induced interfacial charge redistribution, and enhanced catalytic activity collectively improve the performance of the sensor.

In addition to monometallic noble-metal modification, bimetallic functionalization has garnered attention due to the synergistic interactions between various noble metals, promoting enhanced catalytic activity and interfacial charge transfer. Xie et al. fabricated hierarchical flower-like AuPd-decorated WO_3_ nanospheres to detect 3H-2B [20]. The material was fabricated through the uniform anchoring of bimetallic AuPd nanoparticles onto mesoporous flower-like WO_3_ nanospheres, in which the hierarchical architecture provides abundant exposed active sites and interconnected diffusion pathways for gas transport. AuPd-WO_3_ sensor exhibited responses of 84 toward 1 ppm and 400 toward 10 ppm of 3H-2B. Additionally, the sensor demonstrated a low detection limit of 100 ppb, and it exhibited high selectivity with only negligible responses toward common interfering gases. The sensor was able to distinguish LM from *Escherichia coli* and *Staphylococcus aureus* by monitoring the volatile metabolites released during broth cultivation, thereby proving the potential of non-destructive screening of pathogens. The enhanced performance is the result of synergistic effects, which occur between the Au-Pd bimetallic nanoparticles and the hierarchical WO_3_ framework. In comparison to monometallic Au or Pd-modified WO_3_, the bimetallic nanoparticles exhibit higher catalytic activity, which facilitates the adsorption and oxidation of 3H-2B while increasing the concentration of chemisorbed oxygen species on the WO_3_ surface. Simultaneously, the electron transfer between the AuPd nanoparticles and WO_3_ creates a thicker electron depletion layer, resulting in enhanced resistance modulation during gas exposure through electronic sensitization. Finally, the hierarchical flower-like structure minimizes gas diffusion resistance and exposes numerous accessible reaction sites, allowing for fast surface reactions and efficient charge transport.

Some noble metal-functionalized metal oxide semiconductors have been fabricated for the detection of 3H-2B. The sensing performance of these nanomaterials is summarized in Table 3. The noble metals effectively enhance the properties of chemiresistive gas sensors by enhancing catalytic surface reactions and facilitating interfacial charge transfer. Noble metals are able to enhance the adsorption and activation of 3H-2B by chemical and electronic sensitization. These principles lead to higher sensitivity, selectivity enhancement, faster response and recovery kinetics, and reduced operating temperature.

### 3.4. Heterojunction

Apart from the approaches mentioned above, creating heterojunctions is another efficient method for improving the sensing performance of metal oxide semiconductor-based sensors by adjusting the interfacial energy band structure and promoting charge migration from the heterointerface. When two semiconductors with distinct Fermi levels are connected, charge redistribution occurs spontaneously at the interface until an equilibrium is reached [74,75,76]. The charge redistribution process leads to a built-in electric field, as well as the formation of interfacial depletion and accumulation regions. Such interfacial phenomena promote charge separation and enhance resistance variations caused by surface reactions, increasing the sensitivity of the chemiresistive gas sensors [77,78]. Heterojunction engineering makes use of the combined advantages of different semiconductors (e.g., their catalytic activity, carrier transport ability, and affinity toward gas adsorption). This synergistic effect improves the selectivity, rapidity of response, response kinetics, and stability [79,80]. Thus, heterojunction-based materials are in many have been fully studied to detect 3H-2B, which provides a potential method for designing high-performance chemiresistive sensors for LM detection.

Yang et al. designed porous CeO_2_/SnO_2_ nanosheets with rich asymmetric interfacial oxygen sites for highly sensitive sensing of 3H-2B [21]. The coupling of CeO_2_ with the SnO_2_ formed the n-n heterojunction on the porous nanosheet architecture to offer an abundance of accessible surface area and interfacial active sites. Among the samples, CeO_2_/SnO_2_ showed the highest performance, with a response of 637.94 to 50 ppm of 3H-2B and the lowest detection limit down to 137 ppb at an operating temperature of 160 °C. In addition, rapid response and recovery speed, high selectivity against the common interfering gases, and stable repeatability showed that the sensor is well-suited for identifying the volatile biomarker released by LM. The high sensing performance is attributed to the synergistic effect of the CeO_2_/SnO_2_ n-n heterojunction coupled with the asymmetric interfacial oxygen sites. Electrons spontaneously transfer from CeO_2_ to SnO_2_ upon contact, forming an electron-accumulation layer at the interface. The redistribution of the interfacial charge increases the potential barrier and favors the adsorption of oxygen molecules. The asymmetric interfacial Ce-O-Sn sites created active chemisorbed oxygen species that have high reactivity. These species oxidized 3H-2B and accelerated the release of electrons back to the semiconductor conduction band to cause a higher current than that of the pristine SnO_2_. The porous nanosheet structures provided a shortened gas diffusion pathway and more accessible surface reaction sites, further enhancing their response speed and sensitivity. These results showed that local engineering of the interfacial environment of n-n heterojunctions is an effective strategy for charge transport optimization and surface reaction kinetics in chemiresistive sensors.

In addition, n-p heterojunctions have been widely used by regulating interfacial charge redistribution and surface reaction kinetics. Wang et al. synthesized a radial heterostructure of decorating p-Co_3_O_4_ nanoparticles on n-ZnO nanorods by a two-step hydrothermal method [81]. The fabricated sensor showed a response of 550 towards 5 ppm of 3H-2B under an operating temperature of 260 °C. In addition, the sensor showed a low detection limit of 10 ppb, short response and recovery time of 17 s and 8 s, and excellent selectivity to common interfering gases. The improved sensing property is mainly due to the n-p heterojunction formation between ZnO and Co_3_O_4_. Under the influence of the difference in the Fermi levels, electrons flow from ZnO to Co_3_O_4_, and holes flow in the reverse direction until reaching an equilibrium. This process causes an expanded electron depletion layer and a built-in electric field at the interface. The interfacial charge redistribution leads to an enlarged base resistance and electron exchange during the 3H-2B oxidation, which causes an enlarged resistance change and an enhanced response. Additionally, the abundant heterointerfaces and open gas diffusion channels induced by the radial structure promote gas adsorption and surface reaction kinetics.

Many heterostructure sensing materials have been studied to detect 3H-2B. These performances are summarized in Table 4. The built-in electric field formed at the heterointerface promotes carrier separation and enhances the resistance change caused by gas adsorption. On the other hand, the synergistic coupling of different semiconductors introduces complementary features in the catalytic activity, gas adsorption, and charge transport. Therefore, the heterojunction-based sensors generally possess higher sensitivity, higher selectivity, and faster response compared to their single-component counterparts.

Overall, a single-component metal oxide semiconductor presents a fundamental sensing platform for 3H-2B detection due to its simple composition, mature synthesis methods, and reliable sensing performance. Based on this material, heteroatom doping, noble metal modification, and heterojunction engineering have proved to be effective strategies for further improving the sensing performance through tailoring the electronic structure, increasing the number of active sites, and enhancing the gas adsorption and charge migration of the material surface or interface. Recently, studies have gradually integrated multiple modifications, including heteroatom doping with noble metal decoration or heterojunction construction with surface functionalization to achieve synergistic promotion in the performance [88,89]. Although remarkable progress has been achieved in the material design, some challenges remain before the practical implementation is realized. Future research efforts should focus on improving the sensing performance under complex environmental conditions, resisting humidity and interfering gases, and reducing the temperature and power consumption, while simultaneously creating universal material design rules to accelerate the development of highly reliable chemiresistive gas sensors for quick LM detection.

## 4. Practical Applications of Chemiresistive Gas Sensors

### 4.1. Detection of Listeria monocytogenes in Bacterial Cultures and Real Food Samples

The continuous development of chemiresistive gas sensors for 3H-2B has substantially promoted their application in practical LM detection. By targeting this characteristic volatile biomarker, these sensors enable rapid and non-destructive identification of LM without direct bacterial analysis, providing an attractive alternative to conventional microbiological methods. Recent studies have therefore extended their application from standard gas evaluation to bacterial cultures and real food samples, demonstrating considerable potential for food safety monitoring.

**Figure 5:**
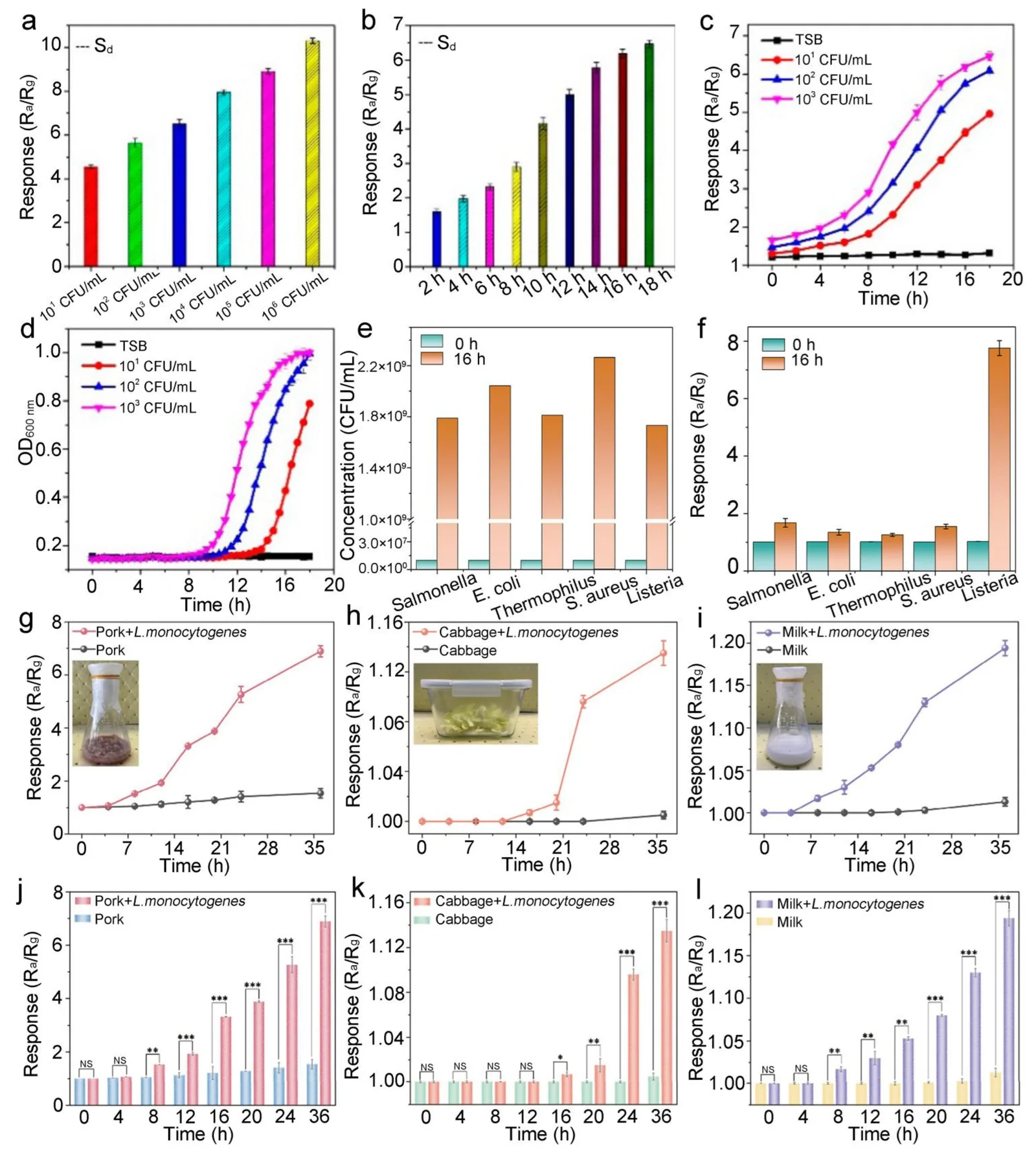
(**a**) Response of the S_d_ (WO_3_-PEO_117_-b-PS_186_) sensor toward LM after 18 h of incubation at 30 °C. (**b**) Sensing performance of S_d_ toward LM (10^3^ CFU mL^−1^) across various incubation durations (2–18 h) at 30 °C. (**c**) Sensitivity tracking of the S_d_ sensor toward 10^1^–10^3^ CFU mL^−1^ LM and a tryptone soy broth control at 2 h intervals (18 h incubation, 30 °C). (**d**) Turbidity monitoring of LM growth (30 min intervals, up to 18 h) in 200 μL wells inoculated with initial bacterial densities of 0–10^3^ CFU mL^−1^ [12]. Adapted with permission from Ref. [12]. Copyright 2017 American Chemical Society. (**e**) Initial and post-incubation (16 h) concentrations of five bacterial species. (**f**) Bacterial gas emission responses of SnO_2_/Au-SAs-0.6% sensor (0 h vs. 16 h incubation) [14]. Adapted with permission from Ref. [14]. Copyright 2024 American Chemical Society. Sensing kinetics of the Au-WO_3_-0.8% sensor toward LM-infected food matrices over time: (**g**) Pork, (**h**) Cabbage, and (**i**) Milk. (**j**–**l**) Corresponding ANOVA significance analysis for storage periods ranging from 0 to 36 h (* *p* < 0.05, ** *p* < 0.01, *** *p* < 0.001, and NS: *p* > 0.05) [23]. Adapted with permission from Ref. [23]. Copyright 2025 American Chemical Society.

Zhu et al. employed highly ordered mesoporous WO_3_ as the sensing material to indirectly monitor viable LM by detecting 3H-2B [12]. The sensor exhibited a clear concentration-dependent response toward LM cultures, with the response increasing from 4.9 to 10.3 as the initial bacterial concentration increased from 10^1^ to 10^6^ CFU mL^−1^ after 18 h of incubation (Figure 5a). More importantly, the sensor was capable of detecting metabolic activity at an early growth stage (Figure 5b). A measurable signal was observed after only 2 h of incubation for cultures with an initial concentration of 10^1^ CFU mL^−1^ (Figure 5c), whereas the conventional turbidity method failed to identify bacterial growth even after 8 h for cultures initially containing 10^3^ CFU mL^−1^ (Figure 5d). This remarkable improvement originates from the detection of metabolically produced 3H-2B. This work demonstrated, for the first time, that chemiresistive gas sensors can achieve rapid and highly selective detection of LM by monitoring its volatile metabolites, providing a practical foundation for subsequent applications in food safety monitoring.

While the above study established the feasibility of chemiresistive gas sensors for rapid LM detection, the ability to discriminate LM from other foodborne bacteria is equally important for practical applications. To address this challenge, Feng et al. developed a single-atom Au-functionalized mesoporous SnO_2_ sensor [14]. The sensor successfully distinguished LM from four other common foodborne bacteria. After 16 h of incubation, when all bacterial cultures reached comparable cell concentrations (approximately 1.7–2.3 × 10^9^ CFU mL^−1^), the sensor produced a response of 7.8 toward LM, whereas the responses to *Salmonella*, *E. coli*, *S. aureus*, and *Thermophilus* were only 1.68, 1.35, 1.55, and 1.26, respectively (Figure 5e,f). Statistical analysis further confirmed the significantly different responses towards LM among different bacterial species (*p* < 0.001). These findings demonstrate that chemiresistive gas sensors are not only for detecting but also for selective identification of LM among other foodborne pathogens, greatly enhancing their practical applicability in the monitoring of food safety.

Recent work extended the application of chemiresistive gas sensors into practical food matrices, where complex volatile backgrounds and variable storage conditions raise higher challenges for reliable detection. Cheng et al. fabricated a Au nanocluster-sensitized WO_3_ sensor and demonstrated nondestructive detection of LM directly in contaminated food products through tracking the concentration level of 3H-2B [23]. The sensor successfully detected the presence of contamination in pork, cabbage, and milk without sample pretreatment. For pork, the sensor response was below 1.1 after the first 4 h of contamination, increased to 1.45 after 8 h of contamination, and reached 7.8 after 24 h, which corresponded to the quick growth of LM in pork (Figure 5g,j). In comparison, the sensor response remained close to the baseline level for uncontaminated pork. For cabbage and milk, a significant difference between the contaminated samples and the control samples was observed after 16 h and 12 h, respectively, but with the overall response being lower than that measured from pork, due to the less favorable growing conditions of LM in these food matrices (Figure 5h,i,k,l). The work shows that monitoring volatile metabolic biomarkers can form an effective strategy for translating the application of chemiresistive gas sensors from laboratory bacterial detection to practical food safety applications.

In summary, chemiresistive gas sensors have been developed since proof-of-concept studies in bacterial cultures up to discrimination of LM from other foodborne pathogens and nondestructive detection in food matrices very recently. These developments show that monitoring the characteristic volatile biomarker 3-hydroxy-2-butanone enables a fast, selective, sample preparation-free strategy for detection of LM, demonstrating the feasibility of chemiresistive gas sensors for rapid and selective LM detection in complex biological and food matrices. Although chemiresistive sensors have demonstrated promising capability for LM detection, direct comparison of their practical performance remains challenging due to the lack of standardized evaluation protocols. Reported studies used different sample matrices, including bacterial cultures and food products, and evaluated sensor performance using different parameters, such as bacterial concentration, VOC concentration, or response value. Moreover, important practical factors, including humidity tolerance, signal reproducibility, long-term stability, and validation against conventional microbiological methods, are rarely systematically investigated. Establishing unified evaluation criteria will be essential for promoting the translation of chemiresistive sensors toward real-world food safety applications.

### 4.2. Portable Sensing Devices and Sensor Integration

Although chemiresistive gas sensors have demonstrated excellent performance in LM detection, translating these sensing materials into practical applications requires the development of portable and integrated sensing systems. By integrating the sensing materials with wireless communication modules, embedded electronics, and intelligent signal processing units, recent studies have greatly promoted the utilization of chemiresistive gas sensors to monitor LM in real-time. These integrated devices not only realize rapid detection but also enable the remote transmission of data and timely alarm, which can further promote the application in food safety monitoring.

**Figure 6:**
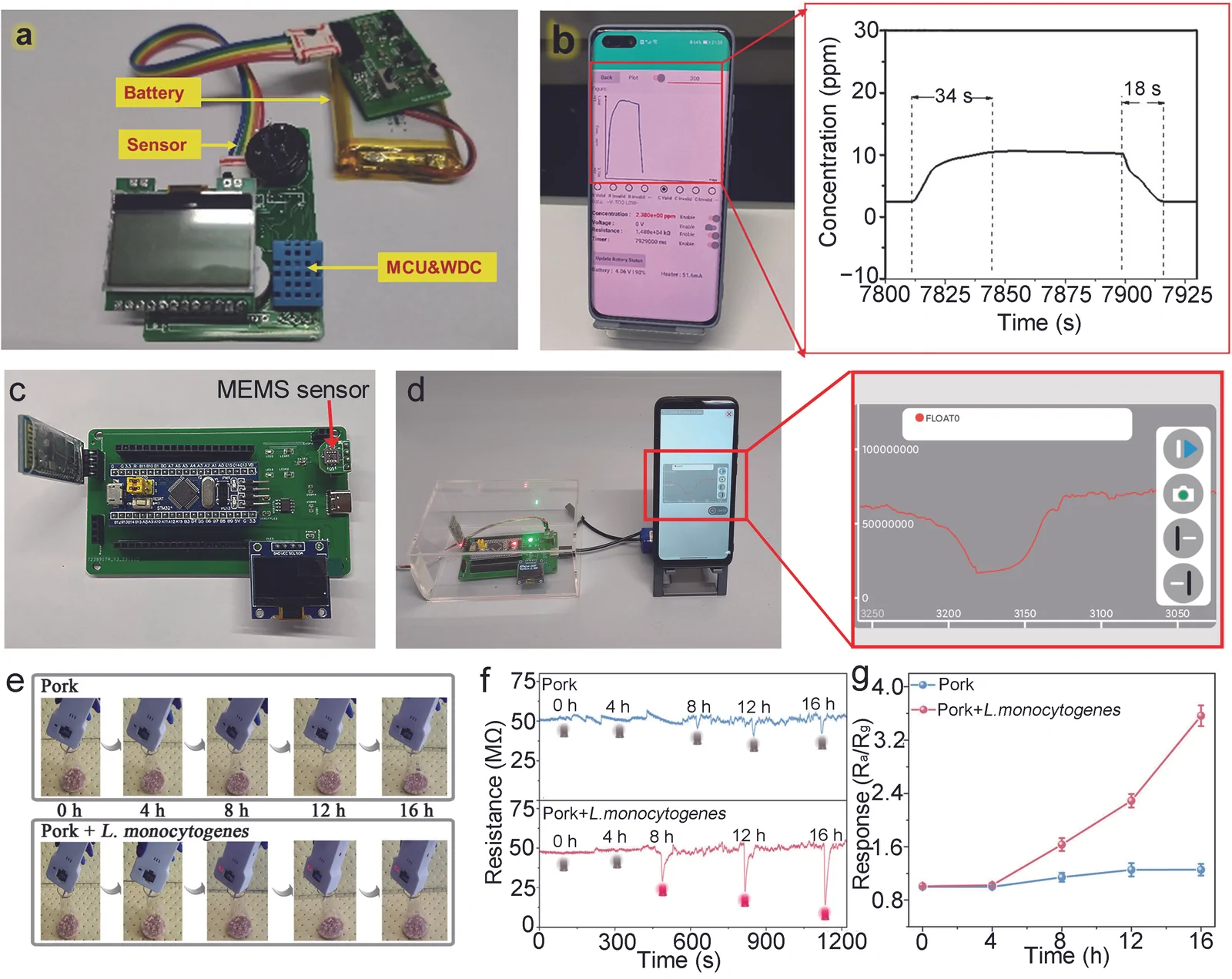
(**a**) Photograph of the Bluetooth-enabled wireless sensing module and smartphone setup. (**b**) Mobile application display during wireless detection of 10 ppm 3H-2B [25]. Adapted with permission from Ref. [25]. Copyright 2021 Wiley-VCH GmbH. (**c**) Photograph of the wireless sensing module. (**d**) Dynamic response–recovery curve of the wireless sensing module toward 1 ppm 3H-2B [14]. Adapted with permission from Ref. [14]. Copyright 2024 American Chemical Society. (**e**) Pork freshness evaluation using a handheld sensor. (**f**) Dynamic response–recovery curves of the handheld sensor for control and *L. monocytogenes*-contaminated pork (0–16 h storage). (**g**) Responses of the pork and pork + *L. monocytogenes* at various storage times (0–16 h) [23]. Adapted with permission from Ref. [23]. Copyright 2025 American Chemical Society.

According to the successful application of the chemiresistive gas sensor in the practical detection of food, the combination of the sensing materials with the wireless communication technologies has been a significant step toward real-time and on-site monitoring. Ma et al. developed a wireless sensing platform by integrating a dual-mesoporous WO_3_/Au gas sensor with Bluetooth-enabled sensing and with a smartphone (Figure 6a), which could realize the monitoring of 3H-2B in real time [25]. The portable system enabled accurate quantification of 10 ppm 3H-2B, with the sensor producing a stable readout of 10.4 ppm within only 34 s and returning to its baseline within approximately 18 s after gas removal (Figure 6b). According to the sensor results measured by the wireless sensing platform, the real-time monitoring results matched the actual concentration of the gases, which ensured the accuracy and stability of the Bluetooth-based signal transmission. This work exhibited that the integration of the chemiresistive gas sensor with the wireless communication module can realize rapid, convenient, and real-time monitoring, which could provide an important basis for the development of portable food safety sensing systems.

Beyond wireless data transmission, existing works further upgraded sensor integration by integrating MEMS technology in combination with intelligent electronic components to build versatile sensing devices. Feng et al. integrated a single-atom Au-functionalized SnO_2_ MEMS gas sensor with an STM32 microcontroller, OLED display, Bluetooth communication module, alarm light, and buzzer to build a compact wireless sensing platform for real-time 3H-2B monitoring (Figure 6c) [14]. Based on the MEMS architecture, the system retained superior sensing properties at low operating temperature (50 °C), yet with low consumption and easy integration. Wireless sensing signals were transmitted to Bluetooth-equipped smartphones or computers, where concentration changes of 1 ppm 3H-2B were monitored (Figure 6d). Equipped with an alarm light and a buzzer, the system triggered simultaneous alarm signals once the preset threshold was exceeded, providing real-time feedback without requiring continuous manual monitoring. Such all-in-one integration significantly improves the practicality of chemiresistive gas sensors and is a crucial step towards intelligent, automatic, and easy-to-apply food safety monitoring.

More recently, the miniaturization of sensing systems has allowed for the use of handheld portable devices for rapid on-site detection. Cheng et al. integrated a MEMS-based Au nanocluster-sensitized WO_3_ sensor with a small-sized compact printed circuit board to form a handheld gas detector with the size of 141.5 mm × 77.5 mm × 35.0 mm [23]. This device can be used directly to analyze volatile metabolites, which are accumulated in the sealed food storage container, without any sample preparation or operation required. Under pork contamination tests, no alarm was activated for uncontaminated samples after 16 h of storage, while LM-contaminated pork triggered both the red warning light and the alarm sound in 8 h. After the sensor was pushed into the container, the sensor resistance dropped quickly to 29 MΩ within 10 s, and its response increased from 1.01 to 3.6 after 16 h, resulting in a good correlation with the bacterial concentration (Figure 6e–g). The findings show that handheld portable chemiresistive gas sensors will provide a rapid, convenient, and nondestructive method for on-site food inspection, which is an important move toward the practical monitoring of food safety.

These studies collectively demonstrate that the integration of chemiresistive gas sensors with wireless communications, smart electronics, and miniaturized hardware has greatly facilitated the development of the transition from laboratory research to practical sensing devices. Compared to conventional gas sensing systems, these integrated platforms allow for real-time data acquisition, remote communication, automatic warning, and user-friendly operation, and thus significantly improve the feasibility of on-site LM monitoring. Although the aforementioned encouraging steps have been taken, most of the portable devices are still at the proof-of-concept level and have primarily been tested under controllable laboratory conditions. Improving the long-term operational stability, lowering power consumption and fabrication costs, and increasing the device’s robustness under complex food storage and transportation environments should be important directions in future attempts.

## 5. Summary and Challenges

Chemiresistive gas sensors have proved an effective strategy for rapid and non-invasive detection of *Listeria monocytogenes* by detecting typical volatile metabolites, especially 3H-2B. As described in this review, recent progress in gas sensing materials like morphology engineering, heteroatom doping, noble metal functionalization, and heterojunction construction has significantly improved the sensor performance. Concurrently, the combination of these gas sensing materials with MEMS technology, wireless communication, and portable electronic systems has facilitated their application to bacterial cultures, food samples, and portable detection devices. These advances show great potential for the application of chemiresistive gas sensors to rapid food safety monitoring and early warning of LM contamination. However, some challenges still restrict practical application.

First, the majority of reported chemiresistive gas sensors use 3H-2B as a unique target biomarker for the detection of LM. While the role of 3H-2B has been widely accepted as a typical volatile metabolite of LM, the production of the biomarker is dramatically affected by bacterial strain, growth phase, nutrient composition, storage temperature, and some other environmental conditions, which may cause variations in signals, thereby affecting the reliability of detection under actual circumstances. Moreover, environmental factors, including humidity, packaging atmosphere, and food-derived background VOCs, may influence the generation, accumulation, and detection of 3H-2B, resulting in variations in sensing signals during practical applications. Therefore, further studies are required to establish standardized detection conditions and improve the robustness of chemiresistive sensors under complex food environments. Advanced metabolomics techniques, especially GC-MS-based volatile metabolomics, are supposed to help to confirm additional typical VOCs and discover time-dependent metabolic profiles with bacterial growth. Based on these results, the development of multi-biomarker-based sensing strategies, rather than a single metabolite as a target, will effectively improve the robustness, specificity, and accuracy of LM detection.

Second, although excellent sensing performance has been achieved under laboratory conditions, complex food matrices, high humidity, and numerous background volatile compounds continue to limit the practical applications of chemiresistive gas sensors. Atomic-level engineering of catalytic active sites using single-atom catalysts and well-defined heterointerfaces is a promising strategy to regulate the gas adsorption behavior and surface reaction pathways for highly selective molecular recognition, while minimizing interference from other volatile compounds. The application of a sensor array with machine learning algorithms is a potential solution that supports practical use. Instead of relying on a single sensing material, pattern-recognition algorithms applied to the response patterns of sensor arrays can generate characteristic response fingerprints, thereby enabling background correction and interference suppression in complex environments. The integration of chemiresistive gas sensors with complementary sensing modalities, such as optical and electrochemical sensing, may provide orthogonal information to improve analytical reliability.

Third, despite many chemiresistive gas sensors having demonstrated extraordinary sensitivity toward 3H-2B, their best performance usually requires an elevated operating temperature, increases energy consumption, and limits continuous portable monitoring. The exploration of high-performance sensing materials that can work efficiently at low or room temperature is a crucial research area. A promising path to this goal is to build efficient catalytic interfaces that reduce the activation energy of surface reactions by rationally regulating catalytic active sites. Emerging materials (e.g., single-atom catalysts, defect-engineered metal oxides, and precisely tuned heterointerfaces) offer new chances to accelerate oxygen activation and gas conversion under mild conditions. Further integration of photo-assisted activation by advanced catalytic materials may offer an effective route to developing low-power, highly sensitive, chemiresistive gas sensors suitable for long-term and portable food safety monitoring.

Fourth, while portable sensing devices have shown successful demonstrations, most existing devices are still at the prototype stage and have been designed for intermittent monitoring rather than long-term continuous monitoring across the food supply chain. The future of such devices would be to further miniaturize and integrate the systems to allow long-term autonomous operation in a practical scenario. Future MEMS, flexible electronics, and low-power integrated circuits will enable smaller, lightweight, low-power devices to be seamlessly integrated into food packaging, smart refrigerators, and cold-chain transport to continuously monitor volatile biomarkers during storage and distribution. Further integration of wireless communication using cloud and data management and edge computing allows for real-time data transmission, remote monitoring, and early warning among multiple storage locations. Emerging self-powered sensing systems using energy harvesting technology could further lower power consumption and potentially lead to a longer operation lifetime, thus promoting the long-term operation of devices rather than frequent battery replacement. These developments would lead to the transition of chemiresistive gas sensors from being detection devices to being part of intelligent monitoring systems.

Integrating microbial metabolomics with chemiresistive gas sensors provides an exciting method for indirect pathogen monitoring. This approach successfully translated discrete microbial metabolic activities into detectable gaseous signals. Though primarily this review is on LM detection through its specific volatile metabolites, the underlying sensing methodologies can easily be extended to other bacteria and fungi with characteristic VOCs. For example, WO_3_ sensors decorated with AuPt nanoclusters have realized ultrasensitive detection for a mold marker, 1-octen-3-ol, as a tool for on-site evaluation of grain safety [90]. The success of such efforts reinforces the generalizability of gas sensing technology across microbial domains. Moving forward, integration of advanced functional materials, sensor arrays, dynamic recognition strategies (e.g., temperature modulation), machine learning data analysis, and metabolomic profiling will probably allow concurrent and real-time monitoring of various volatile markers from multiple pathogens [91]. The combination of chemiresistive gas sensors with complementary detection technologies, such as optical and electrochemical sensing, may provide multidimensional information to improve selectivity and analytical reliability in complex environments. Furthermore, artificial intelligence-assisted data processing and pattern recognition will become increasingly important for extracting meaningful signals from sensor arrays and establishing accurate microbial identification models. These emerging technologies are expected to accelerate the transition of chemiresistive sensing platforms toward intelligent, automated, and versatile microbial monitoring systems. These developments will lay the foundation for integrated microbial surveillance platforms capable of continuous monitoring.

## Figures and Tables

**Table 1:** Gas-sensing performance of single-component metal oxide semiconductor gas sensor toward 3H-2B.

Materials	Temperature (°C)	Concentration (ppm)	Response	Response/Recovery Time (s)	Detection Limit (ppb)	Ref.
WO_3_	290	25	56.1	4/13	100	[12]
NiO	120	50	302	49/52	500	[38]
WO_3_	140	5	32	/	10	[39]
WO_3_	290	20	81	4/9	1000	[40]
WO_3_	240	5	38.8	13/7	100	[41]
ZnO	120	5	328	141/848	1	[42]
Cd_2_GeO_4_	120	10	47	4/1980	100	[43]
WO_3_	205	25	152	25/146	400	[44]
WO_3_	168	20	20.4	2/276	/	[45]

**Table 2:** Gas-sensing performance of heteroatom-doped gas sensor toward 3H-2B.

Materials	Temperature (°C)	Concentration (ppm)	Response	Response/Recovery Time (s)	Detection Limit (ppb)	Ref.
P-WO_3_	300	3	29	26/7	50	[13]
Ce/P-WO_3_	210	10	7.2	9/27	20	[54]
Co-ZnO	260	5	195	53/100	100	[55]
Co-ZnO	305	100	168	1/222	100	[56]
Fe-ZnO	170	100	168.9	2/9	110	[57]
Sn/ZnO	200	100	155.1	1/2	100	[58]
In-ZnO	170	100	830	1/5	2.2	[59]
Cr/WO3	140	5	71.8	27/36	50	[60]

**Table 3:** Comparison of the gas-sensing performance of noble metal functionalization gas sensor toward 3H-2B.

Materials	Temperature (°C)	Concentration (ppm)	Response	Response/Recovery Time (s)	Detection Limit (ppb)	Ref.
Au-SnO_2_	50	2	587.3	10/780	10	[14]
AuPd-WO_3_	250	10	400	8/4	100	[20]
PdAg-ZnO	180	50	157.5	9/6.4	200	[22]
Au-WO_3_	50	0.1	23.2	110/460	8	[23]
Au-WO_3_	175	2.5	18.8	15/45	50	[25]
Ag-WO_3_	240	30	92.7	14/18	/	[67]
Au-ZnO	230	25	174.04	6/7	500	[68]
Pt-SnO_2_	250	10	48.69	11/20	500	[69]
Pd-BiVO_4_	200	10	103.7	12/8	200	[70]
Au-Bi_2_WO_6_	240	50	36.9	13/12	93	[71]
PdRh-Fe_2_O_3_	150	50	148.4	7/10	200	[72]
PtCu-WO_3_	110	10	221.2	9/28	500	[73]

**Table 4:** Comparison of the gas-sensing performance of heterojunction gas sensor toward 3H-2B.

Materials	Temperature (°C)	Concentration (ppm)	Response	Response/Recovery Time (s)	Detection Limit (ppb)	Ref.
CeO_2_-SnO_2_	160	50	637.94	29/172	137	[21]
ZnO-Co_3_O_4_	260	5	550	17/8	10	[81]
Co_3_O_4_-ZnO	260	5	550	/	50	[82]
Cr_2_O_3_-SnO_2_	240	5	50	9/4	20	[83]
CdO-SnO_2_	260	5	45	9/5	20	[84]
ZnO-Al_2_O_3_	300	50	37.2	27/34	1000	[85]
SnO_2_-Al_2_O_3_	120	5	43.3	183/756	100	[86]
ZnIn_2_S_4_-In_2_O_3_	180	50	248.6	18/183	118	[87]

## Data Availability

No new data were created or analyzed in this study. Data sharing is not applicable to this article.
